# Gastroesophageal reflux symptoms in infants in a rural population: longitudinal data over the first six months

**DOI:** 10.1186/1471-2431-10-7

**Published:** 2010-02-11

**Authors:** Robert S Van Howe, Michelle R Storms

**Affiliations:** 1 Department of Pediatrics and Human Development, Michigan State University College of Human Medicine, Marquette, Michigan, USA; 2 Department of Family Medicine, Michigan State University College of Human Medicine, Marquette, Michigan, USA

## Abstract

**Background:**

Increasing numbers of infants are receiving prescription medications for symptoms associated with gastroesophageal reflux. Our aim was to prospectively measure reported gastroesophageal reflux symptoms in healthy term infants for the first six months of life.

**Methods:**

In a prospective cohort study in the rural Upper Peninsula of Michigan, 128 consecutive maternal-infant pairs were followed for six months and administered the Infant Gastroesophageal Reflux Questionnaire Revised (I-GERQ-R) at the one-month, two-month, four-month, and six-month well-child visits.

**Results:**

The I-GERQ-R scores decreased with age. Average scores were 11.74 (SE = 5.97) at one-month, 9.97(4.92) at two-months, 8.44(4.39) at four-months, and 6.97(4.05) at six months. Symptoms associated with colic were greatest at one month of age.

**Conclusion:**

Symptoms of gastroesophageal reflux as measured by the I-GERQ-R decrease with age in the first six months of life in otherwise healthy infants; however the I-GERQ-R may have difficulty differentiating gastroesophageal reflux disease from colic in those under 3 months of age.

## Background

Gastroesophageal reflux (GER) can be a normal physiological process that occurs in healthy infants, children, and adults. GER occurs when gastric contents move into the esophagus as the result of transient relaxations of the lower esophageal sphincter (LES), an abrupt decrease in LES pressure to the level of intragastric pressure unrelated to swallowing, or when the LES tone does not compensate for changes in abdominal pressure[1]. The definition of gastroesophageal reflux disease (GERD), especially in infants, is nebulous. Recently, an expert panel opined that GERD occurred when the reflux of gastric contents is the cause of "troublesome symptoms" and/or complications. "Troublesome" was defined as having "an adverse effect on the well-being" of the patient. The panel did not endorse any objective measures for diagnosing GERD[2].

Another expert panel concluded that there was no evidence to support an empiric trial of acid suppression in infants with symptoms associated with GER[3]. Despite this, many physicians, seeing that parents are concerned or upset by their infant's continual spitting up, are now prescribing both histamine type 2 blocking agents and proton pump inhibitors[4].

The symptoms of GER are nonspecific, making it difficult to distinguish the fussiness, crying, and irritability normally seen in the first months of life from colic, food protein intolerance, or GERD [5-9].

To determine the degree of GER-associated symptoms in a healthy population of infants, we undertook the first prospective cohort study of nonselected infants from birth to six-months of age using the Infant Gastroesophageal Reflux Questionnaire Revised (I-GERQ-R)[10].

## Methods

A consecutive sample of mother-infant pairs who delivered at Marquette General Hospital, a rural referral hospital, was obtained. Exclusion criteria included follow-up with physicians not participating in the study (primarily outside of Marquette, Michigan), gestational age of less than 36 weeks, twins, and admission to the neonatal intensive care unit.

The I-GERQ-R, a validated survey tool, was used to determine the frequency and severity of GER symptoms[10]. The survey was completed by the mother at the one-month, two-month, four-month, and six-month well child visits with the infant care provider. Each question of the survey was evaluated for changes with age.

Comparisons of I-GERQ-R and regurgitation frequency were made. The four items of the I-GERQ-R that addressed symptoms associated with colic (crying, back arching)[6,7] were compared by age to the eight items associated primarily with regurgitation.

Evaluations of changes in scores from visit to visit were made using a t-test. I-GERQ-R scores in those babies currently primarily breastfed were compared to those not primarily breastfed for each postnatal visit. Calculations were performed with SAS version 8.2 (SAS Institute, Cary, North Carolina).

Informed consent was obtained from the mother before entry into the study. This study protocol was approved by the Marquette General Hospital Institutional Review Board.

## Results

A total of 128 mother-infant pairs of the 131 who met the inclusion criteria were enrolled in the study from January 23, 2006 to October 3, 2006. The mothers in our study averaged 27.6 (standard deviation (SD) = 5.5) years old with 1.98 (SD = 1.01) children and 2.1 (SD = 0.59) adults in the home. The babies averaged 39.6 (SD = 1.1) weeks gestational age, birthweight of 3536 (SD = 460) grams, length of 49.9 (SD = 2.5) centimeters, and head circumference of 34.4 (SD = 1.7) centimeters. A review of 2098 infants born at our facility from 1999 through 2001 found that 95.19% were Caucasian, 2.43% Native American, 1.00% mixed race, 0.86% African American, 0.33% Hispanic, and 0.19% Asian American[11]. No shift in the racial/ethnic characteristics of this population has occurred in the intervening years.

**Figure 1:**
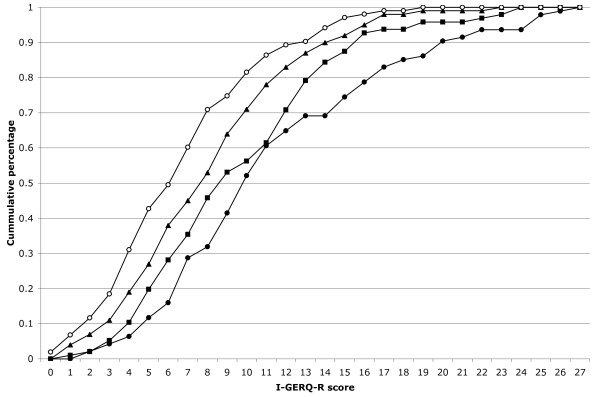
**Infant Gastroesophageal Reflux QuestionnaireRevised (I-GERQ-R) **scores by age (one-month -- solid circle; two-month -- square; four-month -- triangle; six-month -- open circle) represented by cumulative percentage.

The results of the I-GERQ-R surveys by age of visit are given in Table 1 and Figure 1. The leftward shift of the lines with age on Figure 1 indicates a leftward shift of the distribution of I-GERQ-R scores with age consistent with falling scores. The differences in individual patients between I-GERQ-R scores between the one-month and two-month visits was -1.52 (standard error (SE) = 0.49, p < .05), the one-month and four-month visits was -3.63 (SE = 0.56, p < .0001), the one-month and six-month visits was -5.43 (SE = 0.63, p < .0001), the two-month and four-month visits was -1.69 (SE = 0.52, p < .01), the two-month and six-month visits was -3.16 (SE = 0.54, p < .0001), and the four-month and six-month visits was -1.29 (SE = 0.46, p < .01). Each visit resulted in a statistically significant decrease in the I-GERQ-R score from the previous visit. The results of the individual questions of the survey by each visit are shown in Table 2.

**Table 1:** Infant Gastroesophageal Reflux Questionnaire Revised (I-GERQ-R) scores by visit.

Visit	Mean	Standard Error	Median	Interquartile Range
One-month (n = 94)	11.74	5.98	10	7 to 16
Two-month (n = 96)	9.97	4.92	9	6 to 13
Four-month (n = 100)	8.44	4.386	8	5 to 11
Six-month (n = 105)	6.97	4.05	7	4 to 10

**Table 2:** Results of individual questions of the Infant Gastroesophageal Reflux Questionnaire Revised (I-GERQ-R) by age.

Question	One-month	Two-month	Four-month	Six-month
Regurgitation frequency*	1.12(0.09)	1.19(0.09)	1.24(0.09)	0.93(0.08)
Regurgitation volume	1.35(0.07)	1.24(0.07)	1.34(0.07)	1.08(0.08)
Regurgitation discomfort	1.05(0.11)	0.90(0.10)	0.72(0.09)	0.54(0.08)
Refuse feed when hungry	0.24(0.05)	0.21(0.05)	0.28(0.07)	0.24(0.05)
Stop feed when hungry	0.66(0.08)	0.52(0.08)	0.50(0.07)	0.45(0.07)
Cry during/after feed	1.27(0.12)	0.90(0.10)	0.57(0.07)	0.45(0.06)
Cry greater than usual	1.21(0.11)	0.90(0.09)	0.87(0.09)	0.85(0.09)
Cry duration*	0.93(0.09)	0.77(0.08)	0.60(0.07)	0.55(0.06)
Hiccups	2.68(0.08)	2.33(0.10)	1.57(0.10)	1.25(0.08)
Arching	1.13(0.10)	0.86(0.10)	0.72(0.08)	0.57(0.08)
Apnea (awake/struggle)†	0.12(0.05)	0.14(0.05)	0(0)	0.02(0.02)
Turned blue†	0(0)	0.02(0.02)	0(0)	0(0)
Overall severity§	1.37(0.11)	1.23(0.11)	1.18(0.10)	0.88(0.09)

Scores for each question ranged from 0 to 4 except where indicated.

* Score for question ranged from 0 to 3.

† Score for question ranged from 0 to 2.

§Question asked as part of I-GERQ-R, but score not incorporated into I-GERQ-R score.

Mean score (Standard deviation)

Considering a positive score as being an I-GERQ-R score of 16 or greater, 24 of 94 (25.5%, 95%CI = 16.7%-34.4%) of one-month-olds were positive. At two months this dropped to 12 of 96 (12.5%, 95%CI = 5.9%-19.1%). At four months 8 of 100 (8.0%, 95%CI = 3.7%-13.3%) were positive, and at six months 3 of 103 (2.9%, 95%CI = 0%-6.2%) were positive.

The regurgitation frequency remained fairly constant through four months of age (average regurgitations per day: one-month 2.31 (SD = 1.90), two-month 2.19 (SD = 1.89), four-month 2.30 (SD = 1.87)) then dropped at six months of age (average regurgitations per day 1.46 (SD = 1.53), while the I-GERQ-R showed a steady decline with age. When colic-associated scores and regurgitation scores by age were compared, the colic-associated symptoms dropped more quickly over the first two time periods. This is confirmed by a lower percentage of the total I-GERQ-R score contributed by colic-associated symptoms at two months and four months (one-month 35.2%, two-month 32.4%, four-month 29.4%). The rise in the percentage at six months (32.4%) resulted primarily from the drop in regurgitation seen between four and six months of age.

We found no correlation between maternal smoking and colic-associated symptom scores or I-GERQ-R scores. Likewise at each postnatal visit there was no difference in I-GERQ-R scores in those infants who were currently primarily breastfed and those who were not (one-month: difference = 0.56 (95%CI: -2.22, 3.35), t = 0.38, p = .71; two-month: difference = 1.28 (95%CI: -0.72, 3.28,), t = 1.25, p = .21; four-month: difference = -0.73 (95%CI: -2.55, 1.09), t = -0.86, p = .39; six-month: difference = -1.04 (95%CI: -2.76, 0.67); t = -1.25, p = .21).

## Discussion

GER frequency and symptoms, as measured by the I-GERQ-R score, decreased significantly with age. The age pattern of the I-GERQ-R score and regurgitation frequency was different with a steady decline seen in the I-GERQ-R scores while regurgitation frequency was fairly constant until six months of age. The effect of colic-associated symptoms may have undue influence on the I-GERQ-R score in the first two to four months of age.

A drop in the I-GERQ-R score of five or six is considered clinically important, while a three-point drop is considered minimally important[10]. We found a clinically meaningful drop in the average from one month of age to six months of age and a minimally important drop from one month of age to four months of age and from two months of age to six months of age. However, our population was unselected, so lower I-GERQ-R scores on average than those referred for evaluation of GER would be expected. Consequently, any changes in I-GERQ-R scores would be tempered by a floor effect, which is also suggested by the narrowing of the standard error with age.

Despite the high prevalence of GER in the first year of life, this condition has received limited study. In a 1997 study of 948 infants, peak regurgitation (at least one episode of regurgitation a day) occurred in 51% at 0-3 months, in 67% of four-month-olds, and in 5.4% at 10 to 12 months[12].

One-year follow-up on infants who regurgitated daily at 6 to 12 months of age found that none were still regurgitating, but these infants were more likely to report feeding refusal, taking more than one hour to eat a meal, and parents dreading mealtimes[13]. In our population at least one episode of regurgitation was reported in the previous day in 82%, 77%, 83%, and 67% of infants at the one-month, two-month, four-month, and six-month visits, respectively.

In Adelaide, Australia, a peak prevalence (41%) of "spilling" more than half of their feedings occurred between 3 and 4 months of age. The prevalence decreased to less than 5% by14 months of age[14]. In Italy, 7.1% of infants had two or more episodes of regurgitation per day for 3 or more weeks. All had improved at 3-month follow-up[15]. In another Italian study of 2879 infants in the first six months of life, 23.1% had regurgitation and 20.5% had colic[16].

Several studies have focused on irritable infants. Jordan et al. found that 22.8% of irritable infants had a reflux index (RI) (percentage of total time the esophageal pH is less than 4) ≥ 10%[17]. Heine et al. reported that 4.2% of irritable infants under 3 months of age had a RI ≥ 10% while 21.7% of infants three months and older did[18].

Crying duration in infants peaks at six weeks of age with the major concentration between 5 pm and 11 pm with an average of 2.75 hours per day[6,7]. Colic, often defined as three or more hours of crying per day for three or more days per week for longer than two to three weeks, peaks at the same age[19] and has an incidence that ranges from 16% to 20.5%[16,19,20].

Recommended treatment for GER generally consists of parental education, reassurance, and anticipatory guidance[21]. Whether or not an infant is "excessively" irritable or has "troublesome" symptoms is a highly subjective assessment. There is no evidence to indicate that irritability in the absence of regurgitation in infants is related to esophagitis or GER symptoms[18]. The benefit of acid suppression in infants with GER who have either perceived dysphagia or irritability has not been established[3,22].

All infants have some GER,[23] the question is which infants, if any, warrant evaluation and treatment. A recent consensus paper concluded that for infants there is no symptom or symptom complex that is diagnostic of GERD or predicts the response to therapy and there is no consistent correlation between symptoms and an elevated RI or histiological evidence of esophagitis[3]. Environmental smoke exposure has had mixed results with one study finding no association,[14] and two studies finding a positive association[24,25]. Our study found no association between GER symptoms and smoking or breastfeeding. A positive association has been shown between GERD and private insurance,[14] infants with lower birthweights, shorter gestation,[16] and possibly a cow's milk protein allergy[8,17,26]. The most consistent symptom associated with GERD was frequent regurgitation[18,27]. Regurgitating greater than five time per day had a specificity of 70.9%, but a positive predictive value of 22.2%[27].

Adding to the difficulty of identifying and properly diagnosing infants with GERD, there is only poor to fair agreement between finding an RI ≥ 10% and a positive esophageal biopsy[18,28]. While esophagitis may be a significant determinant of long-term outcome,[29] the dangers of an RI ≥ 10% in the absence of esophagitis are unknown.

With the predominance of colic symptoms in the first months of life and a decreased risk of GERD under three-months of age, GERD should not be considered a likely diagnosis until three months of age, and treatment should be reserved for those with histological changes in the lining of the esophagus.

This study was limited by our population: a rural community in which the medical center and a state university are the two biggest employers. Consequently, our population may be more highly educated than other rural populations in the northern Midwest. We had very few of our participants drop out and only a few mothers who refused to participate. We were also limited by lack of access to pH monitoring and pediatric esophogastroduodenoscopy.

Our study highlights the limitations of the I-GERQ-R, which is the only available validated tool to assess GER symptoms in infants. To date, its use has been limited[10,30,31]. Without a clear link between symptoms and disease, the clinical usefulness of the I-GERQ-R may be limited. Expanded experience with the I-GERQ-R is needed, especially at ages when colic-associated symptoms predominate. The values of the I-GERQ-R, or a subset of the items in the I-GERQ-R, that are predictive of GERD may vary with age, and this may need to be taken into consideration when using this tool.

## Conclusion

GER symptoms, as measured by the I-GERQ-R, decrease over the first six-months of life in otherwise healthy infants. Scores in the first months of life may be dominated by colic-associated symptoms.

## Abbreviations

(I-GERQ-R): Infant Gastroesophageal Reflux Questionnaire Revised; (GER): gastroesophageal reflux; (GERD): gastroesophageal reflux disease; (LES): lower esophageal sphincter; (RI): reflux index; (SD): standard deviation; (SE): standard error.

## Competing interests

The authors declare that they have no competing interests.

## Authors' contributions

Both authors contributed to every aspect of the study, with the exception that the statistical analysis was performed by the first author (RSVH). All authors have read and approved the final manuscript.

## About the Authors

RSVH is a Clinical Professor in the Department of Pediatrics and Human Development at Michigan State University College of Human Medicine.

MRS is an Assistant Professor in the Department of Family Medicine at Michigan State University College of Human Medicine and an Assistant Director at the Marquette Family Medicine Residency Program.

## Pre-publication history

The pre-publication history for this paper can be accessed here:

http://www.biomedcentral.com/1471-2431/10/7/prepub
